# The association between the ZJU index and short-term prognosis in patients with acute ischemic stroke: a cohort study

**DOI:** 10.3389/fendo.2026.1899419

**Published:** 2026-08-27

**Authors:** Xiaoyan Zhang, Pan Lu, Yaqi Yang, Li Chen, Zixuan Wei, Lichangtian Jiao, Hongbo Xiao, Jie Wang, Shanbing Hou

**Affiliations:** 1 Rehabilitation Department, The First Affiliated Hospital of Anhui University of Traditional Chinese Medicine, Hefei, Anhui, China; 2 Anhui University of Traditional Chinese Medicine, Hefei, Anhui, China; 3 College of Nursing, Anhui University of Traditional Chinese Medicine, Hefei, Anhui, China; 4 Department of Nursing, The First Affiliated Hospital of USTC, Division of Life Sciences and Medicine, University of Science and Technology of China, Hefei, Anhui, China

**Keywords:** acute ischemic stroke, metabolic biomarker, non-linear association, short-term prognosis, ZJU index

## Abstract

**Objective:**

This study aimed to evaluate the ZJU index—a composite metabolic indicator integrating fasting plasma glucose, body mass index, ALT/AST ratio, and triglycerides—as a predictor of short-term functional outcomes in patients with Acute Ischemic Stroke (AIS) Given the lack of research on this association, the study sought to provide a novel prognostic tool and enhance understanding of metabolic factors in stroke recovery.

**Methods:**

This was a *post-hoc* analysis based on a prospective longitudinal registry database from a single center in Korea. A cohort of 1897 AIS patients was included. The ZJU index was calculated as: FPG (mmol/L) + BMI (kg/m²) + 3 × (ALT/AST ratio) +2 (if female) + TG (mmol/L). Multivariable logistic regression analysis was employed to assess the association between the ZJU index and poor functional outcome (modified Rankin Scale score >2) at 3-month, with sequential adjustments for demographic, clinical, and laboratory confounders. Restricted cubic spline analysis was used to explore the shape of the association, and subgroup analyses were conducted to examine the consistency of the association across different populations.

**Results:**

Multivariable logistic regression revealed a significant negative correlation between the ZJU index and short-term prognosis in AIS patients (after full adjustment for confounders, continuous variable OR = 0.975, 95% CI: 0.952–0.998). Using the first quartile (Q1) of the ZJU index as reference, patients in the second to fourth quartiles (Q2-Q4) had significantly lower risks of poor prognosis. Restricted cubic spline analysis further indicated a significant non-linear negative correlation between the two (Overall *P* < 0.001, Non-linear P = 0.013), presenting a threshold nonlinear trend that the risk of poor functional outcome decreased rapidly with elevated ZJU index and plateaued after reaching a critical cut-off value. Extensive subgroup analyses showed that, except in the diabetes subgroup, this protective association remained consistent across patients of different genders, ages, hyperlipidemia status, smoking status, and history of coronary heart disease.

**Conclusion:**

This study demonstrates that ZJU index is independently associated with short-term functional outcomes in AIS patients, and this association is non-linear. As a composite metabolic indicator, the ZJU index holds potential clinical value for assessing prognosis risk and guiding personalized metabolic management in AIS patients.

## 1 Introduction

Acute Ischemic Stroke (AIS) is the second leading cause of death globally and the primary cause of long-term disability in adults, imposing a substantial disease burden. Epidemiological data indicate that over 12 million new stroke cases occur annually worldwide, with AIS accounting for approximately 85%. The harm of AIS extends beyond its high mortality rate to its significant disabling impact; about one-third of survivors remain dependent in activities of daily living three months post-onset, leading to immense personal, familial, and socio-economic burdens. Therefore, improving patient functional outcomes remains a central clinical challenge (1, 2). Although substantial progress has been made in acute reperfusion therapies, significant heterogeneity persists in long-term functional outcomes (3, 4). Recently, the link between metabolic health and stroke prognosis has garnered increasing attention. Consequently, identifying biomarkers that comprehensively reflect this metabolic status holds significant value for early recognition of high-risk patients and guiding personalized intervention.

Research suggests the association between metabolic disorders and stroke prognosis is underpinned by multi-layered pathophysiological mechanisms (5, 6). Metabolic disturbances such as insulin resistance, obesity, hepatic steatosis, and dyslipidemia are not only crucial risk factors for AIS but may also influence post-stroke recovery by affecting systemic inflammation, endothelial function, and neural repair processes (7, 8). Firstly, insulin resistance and hyperglycemia (reflected in fasting plasma glucose, FPG) can induce the accumulation of advanced glycation end-products, exacerbate oxidative stress and mitochondrial dysfunction, directly damaging neuron and glial cell survival, disrupting the neurovascular unit, and hindering neuroplasticity (9). Secondly, obesity (measured by Body Mass Index, BMI) is not merely a state of energy excess but an active endocrine organ. Through the secretion of various adipokines and inflammatory mediators (e.g., leptin, resistin, TNF-α), it drives chronic low-grade systemic inflammation. This inflammatory milieu can aggravate secondary brain injury post-stroke and suppress neuroregeneration (10). Thirdly, hepatic steatosis and abnormal liver enzymes (assessed by the ALT/AST ratio) are hepatic manifestations of systemic lipid metabolism disorders and insulin resistance. Fatty liver itself exacerbates systemic inflammation and oxidative stress and may influence the central nervous system’s inflammatory microenvironment via the “liver-brain axis” (11). Finally, hypertriglyceridemia is a primary manifestation of lipotoxicity, impairing vascular endothelial function, promoting the progression of atherosclerosis, and potentially interfering with neural repair processes post-stroke by affecting cerebral lipid metabolism and energy supply (12). However, single metabolic parameters (e.g., blood glucose, BMI) have limited predictive value, whereas comprehensive metabolic indices may offer more holistic risk assessment. However, in the acute phase of AIS, the role of these metabolic parameters may be more complex, where BMI and triglyceride composition may also reflect the body’s nutritional reserves and metabolic buffering capacity, thereby affecting the overall prognostic association of the ZJU index.

The ZJU index (Zhejiang University Index) is an emerging, comprehensive tool for assessing metabolic dysregulation, calculated as: FPG (mmol/L) + BMI (kg/m²) + 3 × (ALT/AST ratio) +2 (if female) + TG (mmol/L). This index innovatively integrates the four aforementioned core metabolic dimensions (glucose metabolism, body fat composition, hepatic steatosis, and lipid metabolism) and includes a sex adjustment (13). Prior studies have confirmed that the ZJU index is closely associated with the risk of non-alcoholic fatty liver disease, type 2 diabetes, and cardiovascular diseases (14–16), indicating its effectiveness in reflecting an individual’s overall metabolic burden.

Nevertheless, no study has yet explored the relationship between this composite metabolic index and short-term functional prognosis in AIS patients. Given the potential role of metabolic disorders in stroke pathophysiology and recovery, we hypothesized that ZJU index would present an independent non-linear association with short-term functional outcomes after AIS. This study aims to systematically evaluate the association between the ZJU index and short-term month prognosis in AIS patients through a clinical cohort analysis. The goal is to provide a novel, comprehensive, and easily accessible prognostic prediction tool for clinical practice and to deepen the understanding of the role of metabolic factors in stroke recovery.

## 2 Methods

### 2.1 Data source

**Figure 1:**
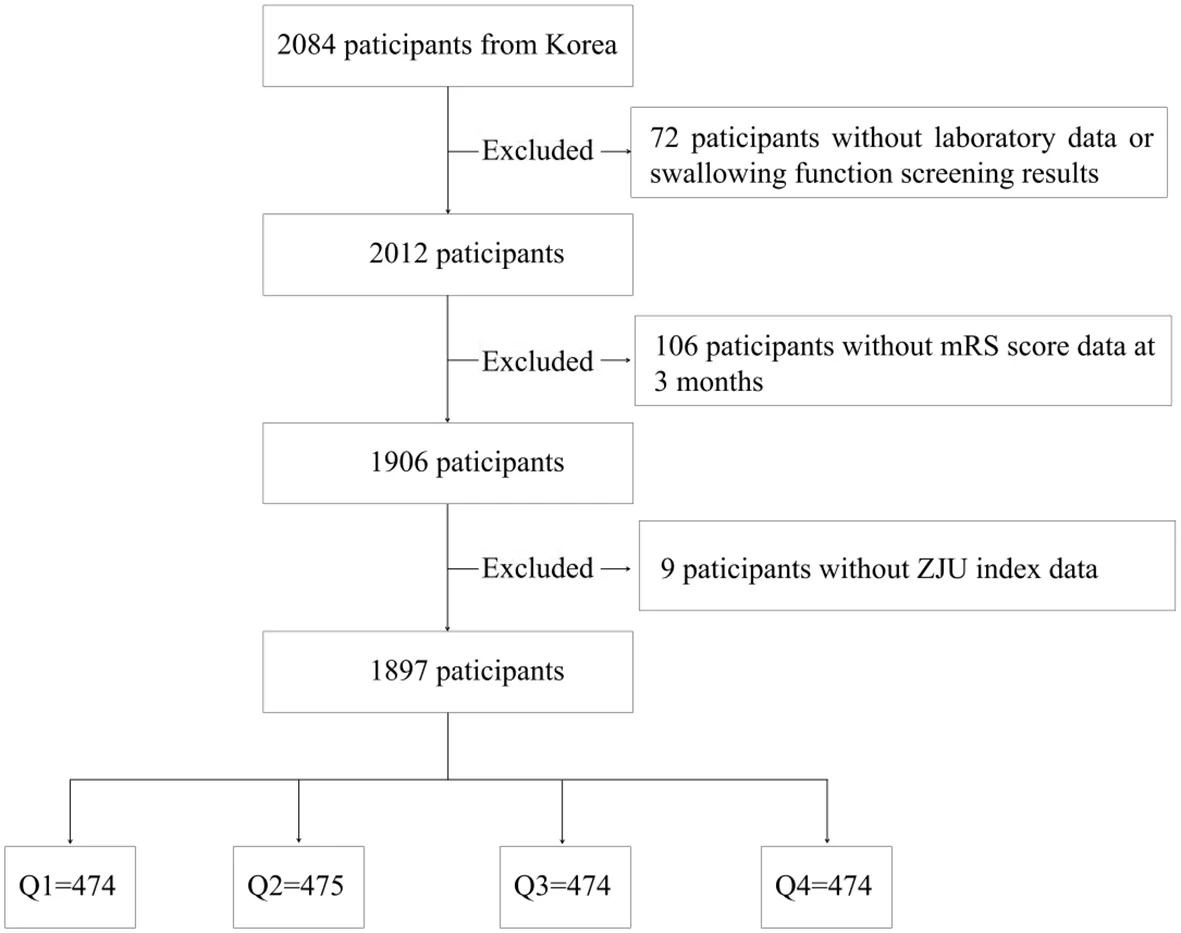
Flow diagram of participant enrollment.

This study is a *post-hoc* analysis based on the database of a 7-year, single-center, prospective longitudinal registry study from Korea. Established in October 2002, this registry was used to screen patients admitted with AIS within 7 days of symptom onset between January 2010 and December 2016 (17). Initial screening identified 2084 patients. Based on the study design, the following exclusion criteria were applied: lack of key laboratory data or swallowing function screening results within 24 hours of admission (n=72); and missing modified Rankin Scale (mRS) score data at 3 months for outcome assessment post-discharge (n=106). Following this screening, 1906 patients met the basic inclusion criteria. To calculate the core exposure variable of this study—the ZJU index—patients missing data for fasting plasma glucose (FPG), body mass index (BMI), alanine aminotransferase (ALT), aspartate aminotransferase (AST), or triglycerides (TG) were further excluded (n=9). Consequently, a final cohort of 1897 patients was included in this analysis (**Figure 1**). The dataset used is publicly shared by the original authors, permitting use for non-commercial scientific investigation and research dissemination with proper citation. The dataset includes 21 baseline demographic and clinical variables, such as age, sex, smoking status, mRS score, BMI, fasting blood glucose (FBG), history of diabetes, hyperlipidemia, and coronary heart disease. Exposure variable: ZJU index, calculated as FPG (mmol/L) + BMI (kg/m²) + 3 × (ALT/AST ratio) + 2 (if female) + TG (mmol/L), a composite marker reflecting overall metabolic status. Primary outcome: Poor short-term functional prognosis, defined as modified Rankin Scale (mRS)>2 at 3-month follow-up; mRS 0–2 represents independent daily living with favorable recovery.

### 2.2 Statistical analysis

Data analysis was performed using SPSS 22.0 and R 4.3.2. Continuous variables conforming to a normal distribution are presented as mean ± standard deviation; otherwise, they are described as median (interquartile range). Categorical variables are expressed as frequency (percentage). For between-group comparisons, the chi-square test was used for categorical variables, and ANOVA or the Mann-Whitney U test was employed for continuous variables based on their distribution. Logistic regression analysis was used to assess the association between the ZJU index and short-term prognosis in AIS patients, with results presented as odds ratios (OR) and their 95% confidence intervals (95% CI). To verify model robustness, three adjusted models were constructed sequentially: Model 1 adjusted for sex and age; Model 2 further added diabetes, hyperlipidemia, smoking, and coronary heart disease; Model 3 additionally included indicators such as white blood cell count, red blood cell count, hemoglobin, red cell distribution width, total cholesterol, high-density lipoprotein, low-density lipoprotein, alkaline phosphatase, albumin, and high-sensitivity C-reactive protein. The selection of confounding factors was based on: (1) identifying basic covariates from previous relevant literature; and (2) including variables that changed the OR by more than 10% upon inclusion. To explore the shape of the association between the ZJU index and short-term prognosis, a restricted cubic spline analysis was performed. Subgroup analyses were conducted by sex, age, diabetes, hyperlipidemia, smoking, and coronary heart disease, with age categorized into four groups using common clinical cut-offs (<60, 60-70, 70-80, >80 years). Interaction analysis was used to test for differences in associations across subgroups. A two-sided *P*-value < 0.05 was considered statistically significant.

## 3 Results

### 3.1 Baseline characteristics

This study included 1897 AIS patients meeting the inclusion and exclusion criteria. Baseline characteristics were analyzed after stratifying patients by ZJU index quartiles (Q1–Q4). Participants comprised 1164 males (61.4%) and 733 females (38.6%). Between-group comparisons revealed that most baseline parameters showed statistically significant differences across quartiles (*P* < 0.05), except for history of smoking, history of coronary heart disease, platelet count, blood urea nitrogen, and creatinine. Details are presented in **Table 1**. Specifically, indicators such as diabetes, hyperlipidemia, white blood cell count, red blood cell count, hemoglobin, triglycerides, low-density lipoprotein cholesterol, alanine aminotransferase, fasting blood glucose, and body mass index showed an increasing trend from Q1 to Q4. In contrast, red cell distribution width, high-density lipoprotein cholesterol, and alkaline phosphatase showed a decreasing trend across quartiles.

**Table 1:** Baseline characteristics of patients included according to ZJU quartile.

Variable	Q1	Q2	Q3	Q4	P
Gender					0.006
Male	311	304	287	262	
Female	163	171	187	212	
Age					<0.001
<60	176	235	241	283	
60~70	192	158	167	151	
70~80	92	76	60	39	
>80	14	6	6	1	
DM					<0.001
No	370	342	337	239	
Yes	104	133	137	235	
Hyperlipidemia					<0.001
No	348	306	293	253	
Yes	126	169	181	221	
Smoking					0.840
No	293	281	291	286	
Yes	181	194	183	188	
Coronaryheartdisease					0.092
No	429	417	425	406	
Yes	45	58	49	68	
Outcome					<0.001
No	293	354	356	351	
Yes	181	121	118	123	
WBC	7.70 [5.92,9.50]	7.48 [6.11,9.27]	7.61 [6.20,9.29]	8.32 [6.98,10.58]	0.003
RBC	4.16 [3.71,4.55]	4.34 [3.94,4.73]	4.42 [4.04,4.79]	4.61 [4.31,5.00]	<0.001
Hb	12.95 [11.48,14.3]	13.70 [12.50,14.90]	13.80 [12.50,14.80]	14.40 [13.20,15.40]	<0.001
RDW	13.3 [12.70,14.10]	13.10 [12.60,13.90]	13.05 [12.60,13.60]	12.80 [12.30,13.40]	<0.001
PLT	212.00 [169.75,257.50]	217.00 [180.00,258.00]	214.00 [182.75,256.25]	170.5 [145.00,200.00]	0.133
TC	170.50 [145.00,200.00]	179.00 [149.00,203.00]	178.50 [151.00,207.25]	193.00 [159.00,225.00]	<0.001
TG	74.50 [54.00,102.00]	91.00 [69.00,115.00]	100.50 [75.00,135.25]	131.00 [92.00,188.00]	<0.001
HDL	45.00 [33.00,58.00]	45.00 [37.00,53.00]	45.00 [36.00,53.00]	42.00 [35.00,51.00]	0.006
LDL	95.00 [69.00,123.00]	104.00 [80.00,128.00]	106.00 [82.00,132.25]	109.00 [83.00,135.50]	<0.001
BUN	16.00 [12.00,21.00]	15.00 [12.00,20.00]	16.00 [13.00,19.00]	16.00 [13.00,19.00]	0.763
Cr	0.089 [0.73,1.11]	0.91 [0.75,1.09]	0.88 [0.74,1.06]	0.88 [0.74,1.10]	0.522
AST	23.00 [19.00,29.00]	22.00 [18.00,29.00]	23.00 [19.00,29.00]	24.00 [19.00,31.00]	0.018
ALT	15.00 [11.00,20.00]	17.00 [12.00,22.00]	19.00 [15.00,26.00]	25.00 [16.50,36.00]	<0.001
ALP	70.50 [58.00,88.00]	70.00 [58.00,86.00]	65.50 [54.00,82.25]	69.00 [56.00,83.00]	0.007
Albumin	4.00 [3.70,4.20]	4.10 [3.80,4.30]	4.10 [3.80,4.30]	4.20 [3.90,4.40]	<0.001
FBG	4.42 [2.07,5.17]	5.17 [4.61,5.83]	5.50 [4.94,6.50]	6.44 [5.39,8.86]	<0.001
hsCRP	0.18 [0.04,0.85]	0.11 [0.30,0.44]	0.11 [0.40,0.39]	0.13 [0.04,0.39]	0.001
BMI	20.09 [18.89,21.64]	22.55 [21.48,23.55]	24.45 [23.29,25.59]	26.62 [24.92,28.34]	<0.001

### 3.2 Multivariate logistic regression analysis

**Figure 2:**
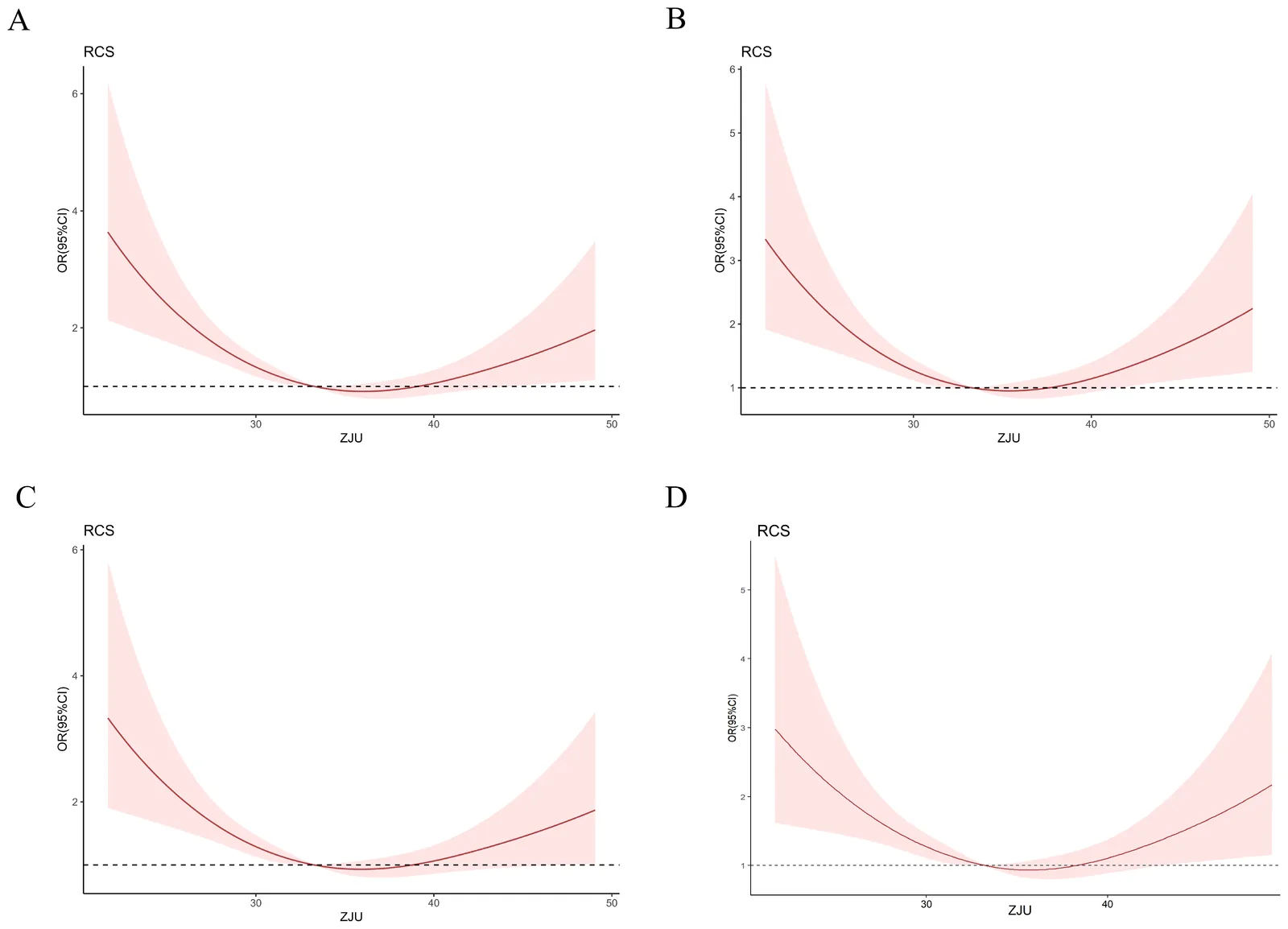
Association between ZJU and short-term prognosis in AIS. **(A)** Unadjusted; **(B)** Model 1; **(C)** Model 2; **(D)** Model 3.

This study employed multivariate logistic regression analysis to assess the association between the ZJU index and 3-month prognosis in AIS patients. The unadjusted model showed a negative correlation between the ZJU index and poor short-term prognosis (OR = 0.965, 95% CI: 0.946–0.985). Using Q1 as the reference, the odds of poor short-term prognosis were significantly lower in Q2, Q3, and Q4, with OR values of 0.553 (95% CI: 0.419–0.730), 0.537 (95% CI: 0.406–0.709), and 0.567 (95% CI: 0.430–0.748), respectively. After further adjustment for sex and age (Model 1), followed by the addition of diabetes, hyperlipidemia, smoking, and coronary heart disease (Model 2), and finally hematological and biochemical indicators (Model 3), the negative association between the ZJU index and poor short-term prognosis remained stable. Analyzing the ZJU index as a continuous variable and adjusting for all confounding factors revealed that for each one-unit increase in the ZJU index, the odds of poor short-term prognosis decreased to 0.975 times the original. Detailed results are presented in **Table 2** and **Figure 2**.

**Table 2:** Logistic regression analysis results for the association between the ZJU and short-term prognosis in AIS.

Vairable	Unadjusted		Model 1		Model 2		Model 3	
ZJU	0.965 [0.946,0.985]	0.001	0.977 [0.956,0.998]	0.032	0.969 [0.948,0.991]	0.006	0.975 [0.952,0.998]	0.037
Q1	Ref		Ref		Ref		Ref	
Q2	0.553 [0.419,0.730]	<0.001	0.592 [0.445,0.788]	<0.001	0.581 [0.435,0.775]	<0.001	0.595 [0.443,0.799]	<0.001
Q3	0.537 [0.406,0.709]	<0.001	0.581 [0.436,0.775]	<0.001	0.565 [0.422,0.756]	<0.001	0.577 [0.427,0.780]	<0.001
Q4	0.567 [0.430,0.748]	<0.001	0.662 [0.495,0.885]	0.005	0.596 [0.440,0.807]	0.001	0.629 [0.459,0.861]	<0.001
P for trend	0.949 [0.925,0.974]	<0.001	0.962 [0.937,0.989]	0.005	0.953 [0.927,0.980]	0.001	0.958 [0.930,0.986]	0.004

Model 1: Gender + Age.

Model 2: Model 1+DM+Hyperlipidemia +Smoking+Coronaryheartdisease.

Model3: Model 2+WBC+RBC+Hb+RDW+TC+HDL+LDL+ALP+Albumin+hsCRP.

### 3.3 Restrictive cubic spline analysis

This study used restricted cubic spline analysis to explore the shape of the association between the ZJU index and short-term prognosis in AIS patients. After full adjustment for potential confounders, the analysis revealed a significant non-linear negative correlation between the two (Overall *P* < 0.001, Non-linear *P* = 0.013). The specific association trend is illustrated in **Figure 2**.

### 3.4 Subgroup analyses

**Figure 3:**
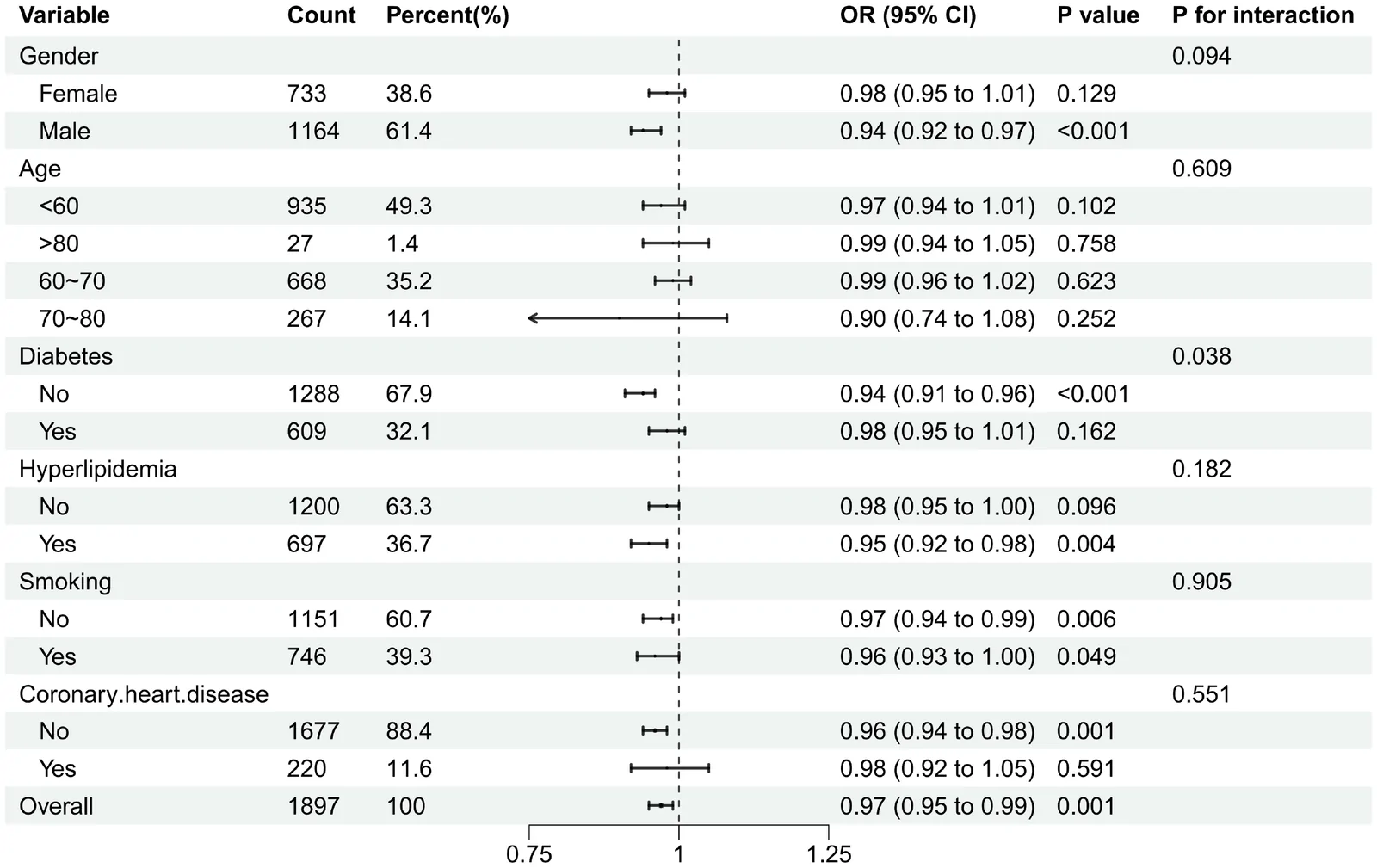
Subgroup analysis for the association between ZJU and short-term prognosis in AIS.

Subgroup analysis and interaction tests were used to assess the association between the ZJU index and short-term prognosis across different populations. Results showed no significant interaction effects in subgroups based on sex, age strata (<60, 60–70, 70–80, >80 years), hyperlipidemia, smoking status, or coronary heart disease; an interaction effect was observed only in the diabetes subgroup. Comprehensive analysis indicated that the negative association between the ZJU index and short-term prognosis remained consistent across subgroups, suggesting the association is robust across populations with different clinical characteristics. Specific results are shown in **Figure 3**.

## 4 Discussion

This study is the first to systematically investigate the relationship between the composite metabolic indicator, the ZJU index, and short-term prognosis in a population with Acute Ischemic Stroke (AIS). The main findings are as follows: Multivariate logistic regression analysis revealed a significant negative correlation between the ZJU index and the short-term prognosis of AIS patients, and this association remained robust after full adjustment for demographic, clinical, and laboratory confounding factors. Restricted cubic spline analysis further indicated a significant non-linear negative correlation between the two. Extensive subgroup analyses showed that, except in the diabetes subgroup, this protective association remained consistent across patients of different genders, ages, hyperlipidemia status, smoking status, and coronary heart disease history, suggesting its generalizability and reliability.

The ZJU index is composed of fasting plasma glucose (FPG), body mass index (BMI), the liver enzyme (ALT/AST) ratio, and triglycerides (TG), its value lies in integrating of multiple independent metabolic risk markers into a simple, quantifiable composite indicator, which has been proven effective in predicting risks for diabetes, non-alcoholic fatty liver disease, hypertension, and more. Researches show that the ZJU index can effectively identify high-risk groups of diabetes, and has a strong positive correlation with the incidence rate of diabetes (18, 19).As an indicator that integrates liver enzymes (ALT/AST) and metabolic parameters (FPG, TG, BMI), the ZJU index has been shown to be significantly correlated with the severity of NAFLD diagnosed by ultrasound, and NAFLD patients with higher ZJU index have a significantly increased risk of liver fibrosis progression, demonstrating good diagnostic performance in community screening (14, 20). Other studies have also shown that the ZJU index is closely related to poor blood pressure control (in a non-linear dose-response pattern), cardiovascular disease (CVD) risk and mortality, U-shaped all-cause mortality, polycystic ovary syndrome, and metabolism related kidney disease in hypertensive patients (16, 21–23).

Given the previously reported associations between the ZJU index and diverse metabolic risks, the protective association observed in this AIS cohort warrants further investigation. Higher BMI and better nutritional status are generally associated with favorable outcomes across various disease settings (24, 25). In AIS, the BMI and TG components of the ZJU index may serve as proxies for metabolic reserve, potentially supporting neurorepair and recovery (26–28), while FPG and ALT/AST may further modulate this association through stress-related energy mobilization and hepatic metabolic adaptation (29, 30). Thus, a lower ZJU index may reflect underlying malnutrition, sarcopenia, or frailty. In the hypercatabolic acute phase of AIS, patients with insufficient metabolic reserve (manifested as low BMI and low TG) may have impaired ability to withstand heightened metabolic stress, leading to poorer recovery. This aligns with the malnutrition-inflammation-atherosclerosis (MIA) paradigm, which emphasizes the prognostic role of nutritional status (31). Supporting this, malnutrition, low BMI, and frailty have been consistently linked to adverse post-stroke outcomes, and a lower Geriatric Nutritional Risk Index (GNRI) predicts poor 6-month prognosis in AIS patients (32–35). Collectively, these observations indicate that the ZJU index may serve as an indirect indicator of nutritional reserve via its BMI and TG components, contributing to its prognostic value. In addition, educed metabolic reserve may explain the link between low ZJU index and poor AIS prognosis. In the acute phase, heightened metabolic stress demands substantial energy substrates for neurorepair and immune/inflammatory responses. Patients with greater reserve (higher BMI and TG) may have superior “metabolic buffering capacity” to tolerate this stress, while those with lower reserve (low BMI and TG) show poorer resilience and outcomes. This hypothesis aligns with the obesity paradox, suggesting that adequate energy reserves may be prognostically more critical than metabolic normalization in acute settings.

This study demonstrates that the ZJU index, despite its established role in capturing clustered metabolic risk factors, exhibits an inverse and non-linear association with poor short-term prognosis in AIS patients. This may be attributable to the BMI and TG components of the index, which in the acute phase may serve as proxies for nutritional reserve and metabolic buffering capacity rather than mere markers of metabolic disturbance. A low ZJU index may thus indicate malnutrition or frailty, consistent with the obesity paradox. As a simple and readily available composite measure, the ZJU index may facilitate early risk stratification by identifying patients with diminished physiological reserve. However, given the observational design and lack of direct nutritional and stroke severity measures, these findings warrant cautious interpretation and further validation in prospective studies.

## 5 Study strengths and limitations

The strengths of this study include its prospective registry-based design, relatively large sample size, systematic adjustment for multiple confounders, and comprehensive subgroup and nonlinear analyses. Several limitations should also be acknowledged. First, the single-center observational design limits generalizability and precludes causal inference. Second, residual confounding cannot be excluded, particularly due to the absence of key prognostic variables such as stroke severity (e.g., NIHSS), reperfusion therapy, and infarct volume. Third, selection bias may have arisen from excluding patients with missing data. Fourth, only 3-month outcomes were assessed; long-term prognosis, recurrence, and mortality remain unexplored. Consequently, the observed association should not be interpreted as a protective effect of metabolic dysregulation; rather, the ZJU index may serve as a composite marker of nutritional and metabolic status. Its prognostic utility requires prospective validation in multicenter studies incorporating comprehensive nutritional assessments (e.g., GNRI), sarcopenia screening, and stroke severity metrics.

## 6 Conclusion

This study demonstrates that ZJU index is independently associated with short-term functional outcomes in AIS patients, and this association is non-linear. As a composite metabolic indicator, the ZJU index holds potential clinical value for assessing prognosis risk and guiding personalized metabolic management in AIS patients.

## Data Availability

The original contributions presented in the study are included in the article/supplementary material. Further inquiries can be directed to the corresponding authors.
